# Improving predictive performance in incident heart failure using machine learning and multi-center data

**DOI:** 10.3389/fcvm.2022.1011071

**Published:** 2022-10-18

**Authors:** František Sabovčik, Evangelos Ntalianis, Nicholas Cauwenberghs, Tatiana Kuznetsova

**Affiliations:** Research Unit of Hypertension and Cardiovascular Epidemiology, KU Leuven Department of Cardiovascular Sciences, University of Leuven, Leuven, Belgium

**Keywords:** heart failure, incidence, machine learning, prediction model, multi-center data

## Abstract

**Objective:**

To mitigate the burden associated with heart failure (HF), primary prevention is of the utmost importance. To improve early risk stratification, advanced computational methods such as machine learning (ML) capturing complex individual patterns in large data might be necessary. Therefore, we compared the predictive performance of incident HF risk models in terms of (a) flexible ML models and linear models and (b) models trained on a single cohort (single-center) and on multiple heterogeneous cohorts (multi-center).

**Design and methods:**

In our analysis, we used the meta-data consisting of 30,354 individuals from 6 cohorts. During a median follow-up of 5.40 years, 1,068 individuals experienced a non-fatal HF event. We evaluated the predictive performance of survival gradient boosting (SGB), CoxNet, the PCP-HF risk score, and a stacking method. Predictions were obtained iteratively, in each iteration one cohort serving as an external test set and either one or all remaining cohorts as a training set (single- or multi-center, respectively).

**Results:**

Overall, multi-center models systematically outperformed single-center models. Further, c-index in the pooled population was higher in SGB (0.735) than in CoxNet (0.694). In the precision-recall (PR) analysis for predicting 10-year HF risk, the stacking method, combining the SGB, CoxNet, Gaussian mixture and PCP-HF models, outperformed other models with PR/AUC 0.804, while PCP-HF achieved only 0.551.

**Conclusion:**

With a greater number and variety of training cohorts, the model learns a wider range of specific individual health characteristics. Flexible ML algorithms can be used to capture these diverse distributions and produce more precise prediction models.

## Introduction

A major burden of modern society is progressive increase in age-associated disorders such as cardiovascular (CV) diseases. Due to population aging and unhealthy lifestyle, the prevalence of heart failure (HF) in low- to middle-income countries will rise by 50% in the next 5–10 years (1). According to the World Health Organization, proper early risk stratification and management could help reduce the burden of this chronic disorder.

There are currently a number of clinical risk scores indented for specific populations and risk groups (2–6). For example, the Pooled Cohort Equations to Prevent HF (PCP-HF) score is recommended to use for 10-year incident HF prediction in a general population (7). Most of the recommended scores are, however, based on a linear model and might therefore lack the specificity and sensitivity in certain subgroups. The wide variety of scores may also cause slow adoption in clinical practice.

The use of advanced analytic techniques such as machine learning (ML) might improve the predictive performance of models by employing a higher number of interrelated and non-linear features. In addition, training ML models on a wide range of patient groups might create tools generally applicable in different settings. Recently, the number of publications applying ML for both prognosis and diagnosis of CV disease sharply increased, with 85% of these decided in favor of ML as opposed to traditional linear methods (8). The most popular choices include tree-based boosting and bagging methods, such as survival gradient boosting (SGB) and random survival forests (RSF) (9). However, a substantial obstacle in the adoption of ML in CV risk prediction, including HF, is the lack of adequate external validation in a large number of individuals with varied characteristics, as well as the ability to exploit flexible models when trained in various populations using a large number of relevant features (8).

Therefore, we proposed to test predictive model performance in a spectrum of train and test populations systematically instead of selecting a single derivation and validation cohort. Similarly, choosing an appropriate evaluation strategy (internal, external, etc.) is crucial (10). Thus, we additionally evaluated the influence of obtaining more diverse training data (multi-center) on the predictive performance of incident HF prediction models.

## Objectives

The main objective of our analysis was to evaluate the predictive performance of the incident HF risk prediction models in the general population. Specifically, we compared the predictive performance of a linear model (CoxNet), non-linear model (SGB), currently used HF risk score (PCP-HF), and a stacking method (combining prediction of tested models). We also evaluated the predictive performance of models when trained on multiple heterogeneous cohorts (multi-center) rather than on single cohort. In addition, we reported features selected by the models and assessed their achieved predictive performance for the given number of features.

## Methods and materials

**Figure 1:**
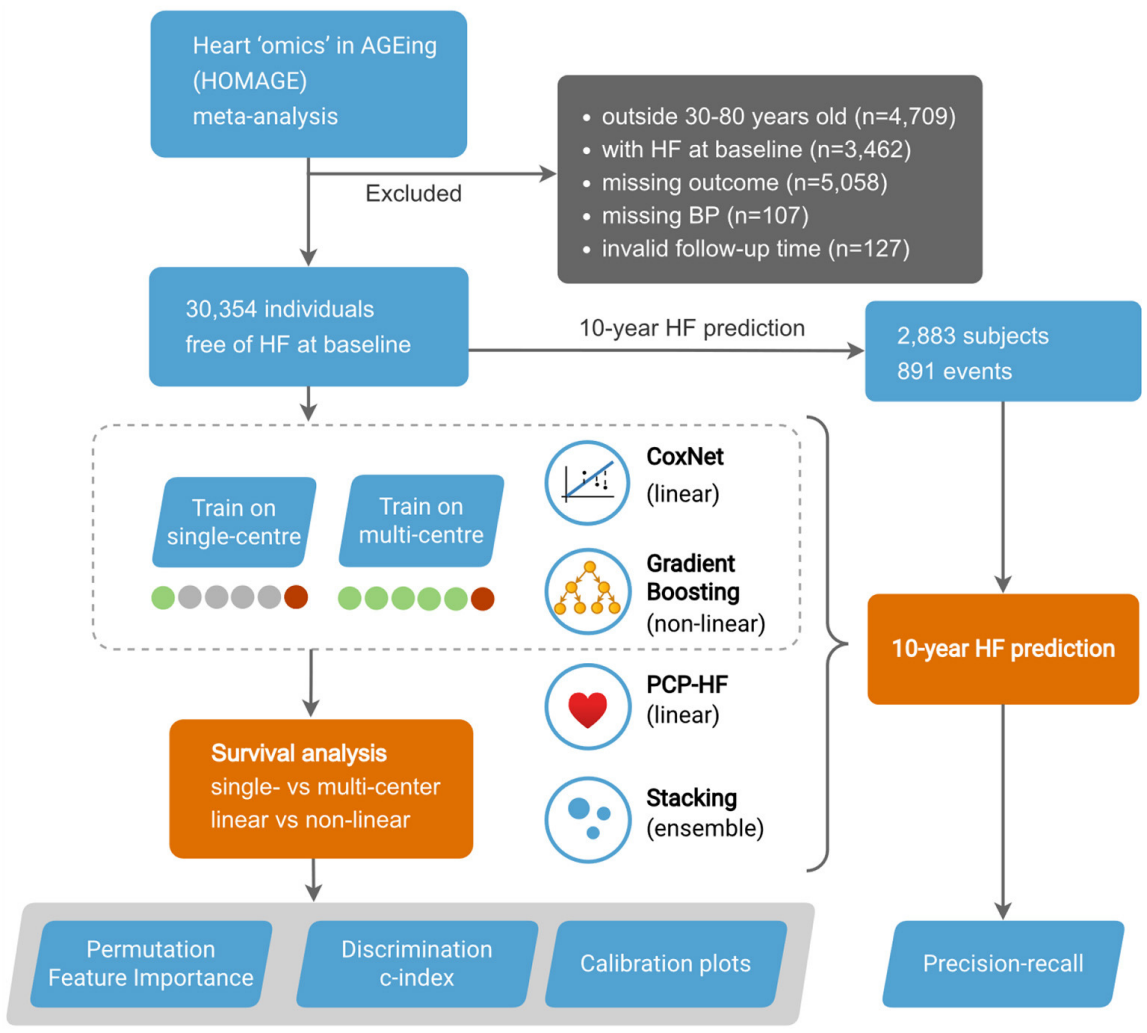
Study design.

Study design is outlined in Figure 1.

### Cohorts

In our analysis, we included 6 cohorts from the Heart “Omics” in Aging (HOMAGE) meta-data—the Anglo-Scandinavian Cardiac Outcomes Trial (ASCOT), the Flemish Study on Environment, Genes, and Health Outcomes (FLEMENGHO), the Health Aging and Body Composition (Health ABC), HVC database, Valutazione della PREvalenza di DIsfunzione Cardiaca asinTOmatica e di scompenso cardiaco (PREDICTOR), and the Prospective Study of Pravastatin in the Elderly at Risk (PROSPER). The final dataset included 33 features consisting of clinical (e.g., medical history, HR, SBP), biochemical (e.g., blood glucose, creatinine), and ECG (e.g., duration QRS) variables. For a complete list of included features, see Supplemental Table 1. The unfiltered input data of the individuals consisted of 43,817 individuals. We removed participants younger than 30 and older than 80 years old (n = 4,709), with HF diagnosis at baseline (n = 3,462), with missing outcome (n = 5,058), with missing blood pressure measurements (n = 107), and with invalid follow-up time (n = 127). The final study population included 6 cohorts consisting of 30,354 subjects.

By sending anonymized data, the contributing partners confirmed that their study complies with good clinical practice (Helsinki Declaration), that all participants provided written informed consent, and that at the time of its conduct the study conformed to national regulations on clinical research in humans and on the protection of privacy. The HOMAGE database was described in depth elsewhere (11, 12).

### Outcome of interest

The primary outcome of interest in this study is incident non-fatal HF, defined as HF hospitalization. The specificities of non-fatal HF in each of the cohorts were described elsewhere (11, 12).

### ML algorithms

We evaluated the predictive performance of the following two models: a flexible survival ML model (Survival Gradient Boosting, SGB) and a linear Cox proportional hazard model-CoxNet. SGB is a non-linear machine learning method based on training regression trees with the objective of optimizing Cox partial likelihood. CoxNet is a standard linear Cox proportional hazard model, regularized by both L1 and L2 norms. As an alternative, we also employed the stacking method, consisting of the above-mentioned CoxNet and SGB, together with PCP-HF score and unsupervised Gaussian mixture model. The stacking method works in two layers, the output of the first layer of base learners is the input of the second “meta” layer, consisting of another model. For CoxNet, the features were standardized. We used the Optuna library with the tree-structured parzen estimator to optimize the model hyperparameters on an internal validation set. For ML pipelines, we used the scikit-learn and scikit-survival Python libraries. The code of the analysis is available online^1^.

### Model evaluation and statistical analysis

The discrimination of the models was evaluated using the c-index. Predictive performance was evaluated iteratively, with each cohort serving as a test set (external cohort validation). In each iteration, the multi-center models were trained on the remaining 5 cohorts (1 cohort test), while the single-center models were trained on a single cohort. The final predictive performance was obtained by evaluating the merged predictions and, in the case of single-center models, averaged. The goal was to compare the predictive performance of models trained in limited data (single-center) and those trained in a more comprehensive sample (multi-center) (Figure 1).

All optimization/training pipelines were run 10 times, each time with a different random seed, producing 10 slightly different models. To provide a more stable prediction, each prediction was a mean of predictions of these 10 models. To obtain a more balanced estimate, samples were weighted inversely to the proportions of sizes of their corresponding cohorts. Therefore, in effect, each cohort had the same influence on predictive performance. We performed the two-sample Kolmogorov-Smirnov test to evaluate the statistical significance of the difference in c-index of the models. We also performed a feature importance analysis to illustrate which features were important for specific models and, therefore, might carry clinically useful information.

In addition, we used the 10-year binary endpoint to evaluate the models in clinically relevant precision-recall (PR) analysis (Figure 1). PR analysis is more sensitive to differences in false positives and thus better captures the practical aspect of clinical decision making. In the process of binarizing the outcome, subjects censored before the endpoint were removed. The predictions in the calibration plot were divided into bins on the x axis according to the predicted probability of an event, and this probability was put into comparison with empirical event incidence in the given bin on the y axis. 95% confidence intervals were calculated from 100 bootstrapped test scores, thus quantifying uncertainty on the unseen data (assuming the training data were given).

## Results

### Study population

The dataset consisted of 30,354 individuals (mean age 66 ± 9 years, 66.57% male), free of HF at the baseline. For detailed cohort characteristics and missing values, see Supplemental Tables 1, 2, respectively. During a median follow-up of 5.40 years (IQR 4.28–6.52), 1,068 individuals experienced at least one non-fatal HF event (6.46 events per 1,000 person-years). For a detailed overview of the outcome statistics, see Supplemental Table 3. In this study, we also evaluated the HF prediction at the endpoint of 10 years. After removing censored subjects, we obtained 2,883 subjects (891 events) for the 10-year prediction (Figure 1).

### Comparing predictive performance of single-/multi-center and linear/non-linear models

**Figure 2:**
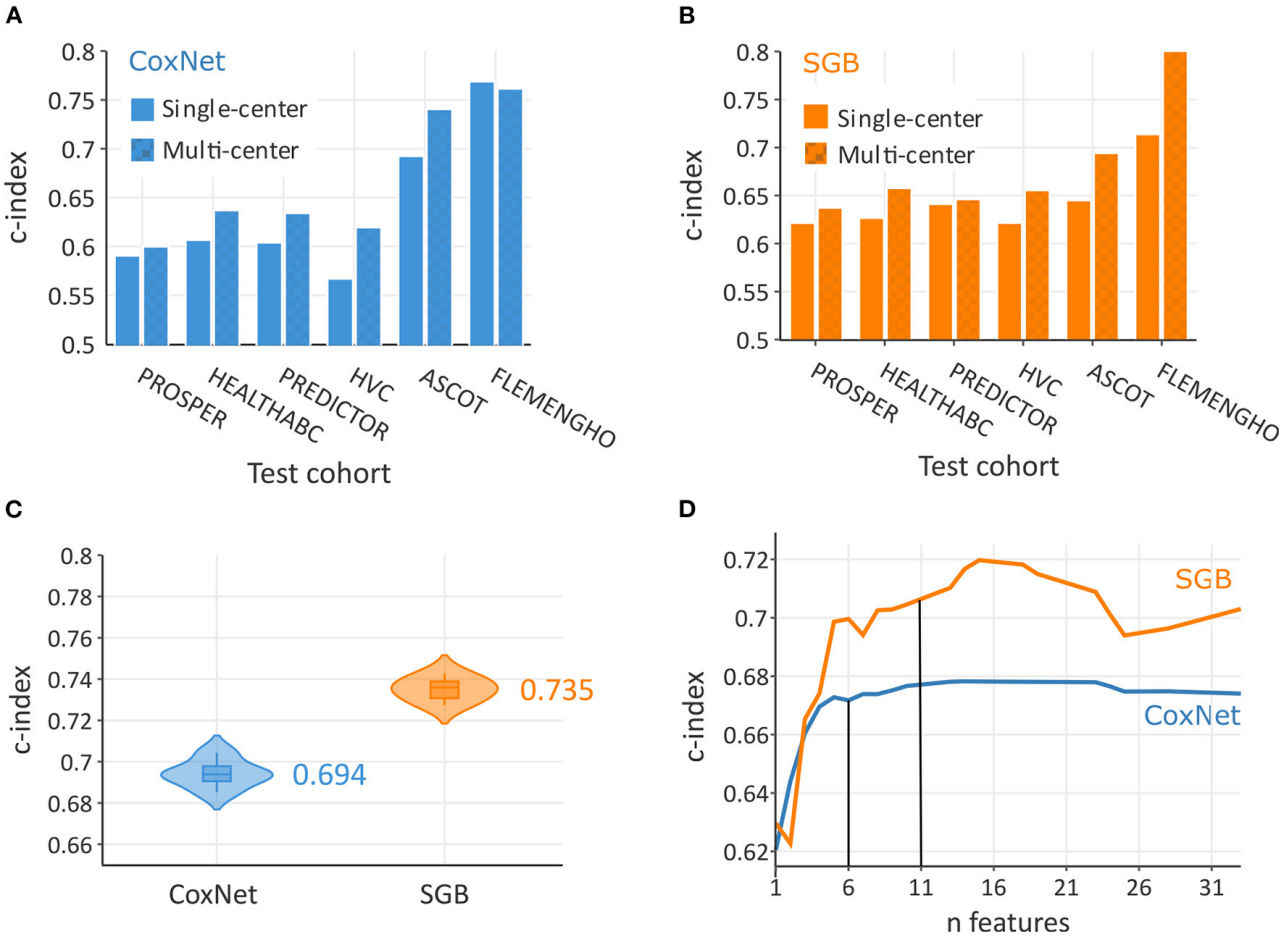
Predictive performance increased with multi-rather than single-cohort training data both in CoxNet **(A)** and survival gradient boosting (SGB) **(B)**. On the pooled dataset, SBG achieved a greater overall c-index than CoxNet **(C)**. In terms of number required features to achieve maximum predictive performance, SBG required 11 features and CoxNet 6 features **(D)**.

As illustrated in Figures 2A,B, multi-center models systematically outperformed single-center models. For example, when testing on the FLEMENGHO cohort, multi-center SGB achieved c-index of 0.812, while only 0.714 in a single-center setting. Overall, the c-index in the pooled population (Figure 2C) was significantly higher (*P* < 0.0001) in non-linear SGB (0.735; 95% CI: 0.728–0.742) than in linear CoxNet (0.694; 95% CI: 0.686–0.704). These c-index values corresponded to risk discrimination between all individuals, and, therefore, represent the situation of a heterogeneous population. The calibration plot in Supplemental Figure 1 showed good calibration of both methods, but SGB showed a certain overestimation of risk in some individuals with lower risk.

### Important features for incident HF prediction

**Figure 3:**
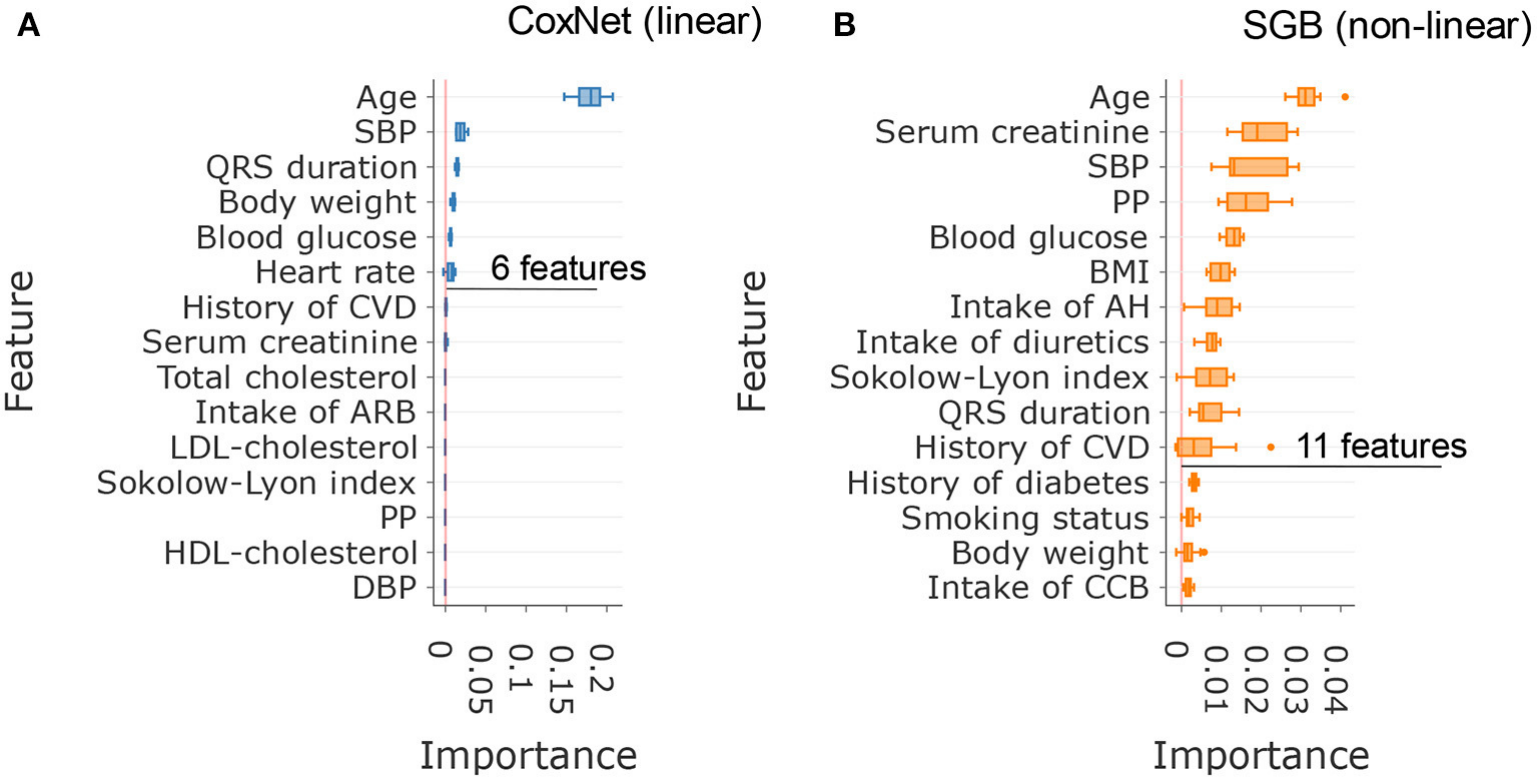
Comparison of the most important features in CoxNet **(A)** and survival gradient boosting **(B)**.

In this analysis, SGB achieved maximal predictive performance with about 11 features, whereas CoxNet exploited fewer features and achieved its maximal predictive performance with only 6 features (Figure 2D). Figure 3 showed the importance of the permutation obtained from changes in predictive performance when supplied with shuffled input features. The CoxNet predictive model relied heavily on age, with a smaller influence of several other predictors, including SBP, QRS duration, and body weight. On the contrary, SGB employed a wider range of features beyond age, including serum creatinine, blood pressure, blood glucose, BMI, ECG features, antihypertensive treatment, and CV disease history.

### Precision-recall of 10-year HF prediction using novel and traditional scores

**Figure 4:**
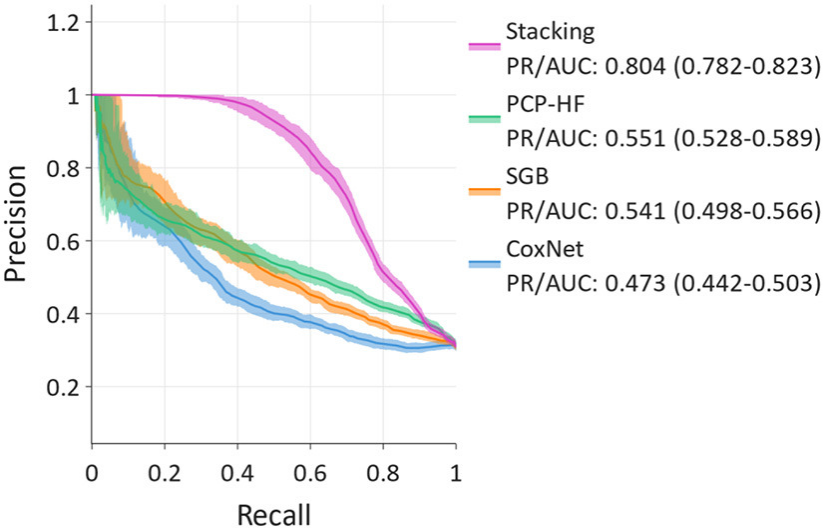
Precision-recall curves for 10-year prediction showing stacking, Survival Gradient Boosting (SGB), CoxNet, and PCP-HF. Stacking exhibits the best discrimination.

For predicting the 10-year risk of incident HF, the stacking method, combining the SGB, CoxNet, Gaussian mixture, and PCP-HF models, achieved the best discrimination in the PR analysis (Figure 4), with PR/AUC 0.804 (95% CI: 0.782–0.823). SGB and PCP-HF performed similarly with PR/AUC of 0.541 (0.498–0.566) and 0.551 (0.528–0.589), respectively. CoxNet achieved PR/AUC of 0.473 (0.442–0.503).

## Discussion

### Predictive performance of ML and linear methods in incident HF prediction

In the line with previous studies focusing on the comparison of ML methods with linear-based scores (9, 13–15), our study supported the findings that the ML model (SGB) detected more subtle patterns for incidence HF prediction than the linear CoxNet. Simultaneously, the predictive performance of ML algorithms varies dependent on a given task. Given the heterogeneity of predictive performance, stacking is a suitable tool capable of increasing predictive performance by combining the output of several estimators (models) using another ML meta-model. In our study, the stacking model, consisting of SGB, CoxNet, Gaussian Mixture, and the PCP-HF score, outperformed all tested models for 10-year incident HF prediction.

The full capacity of ML in the deep characterization of clinical phenotypes and therefore the delivery of personalized medicine is still unknown. The potential of using ML in HF prediction (and CV more broadly) depends strongly on the depth and volume of representative data points available. For example, Balabaeva et al. reported that temporal features increased the predictivity of ML in assessment of symptomatic HF prognosis (16). Therefore, integration of the temporal domain into prediction models might be the next logical step in the application of ML in CV/HF risk stratification. Using two or more data points could help extrapolate the rate of change in one's health status more precisely.

### Multi-center data increased predictive performance in HF prediction

The collection of representative data is crucial for success in the development of reliable risk stratification tools. Our analysis showed that models trained on a combination of cohorts outperformed models trained on a single cohort (Figures 2A,B). These results advocated for use of large and representative training data for developing robust prediction tools. Indeed, flexible ML models, such as gradient boosting, require a large training sample to cover the full feature space. However, clinical data use must be in accordance with data privacy and might struggle with pitfalls in data sharing.

### Dataset shift and multi-center data

One of the main reasons for maximizing the volume of training data is to overcome the so-called “dataset shift” problem. This problem relates to a shift in the distribution of derivation and validation (test, real-world) cohorts (17). It occurs for various reasons, such as differences in population characteristics, hospital procedures, selection bias, etc. The dataset shift manifests itself as a decrease in predictive performance when the model is tested on an unseen population, and therefore the external validation is crucial in estimating the generalization of the model. One straightforward and effective approach to combating the dataset shift is to train flexible ML models on a large volume of representative data (17). An example of difficulties originating from the lack of robust predictive models trained on such diverse data is the need to recalibrate current HF and/or CV risk scores for each tested population (5). With a greater number and variety of training samples, ML models can learn a wider range of health profiles. Flexible ML algorithms, such as gradient boosting, can thus be used to capture these diverse distributions and produce more precise, personalized models for the prediction of adverse events. For instance, the ML model that captures personalized characteristics (e.g., ethnic differences) achieves better predictive performance compared to the traditional one (9).

### Data sharing issues and regulation

As outlined above, to effectively evaluate the benefit of ML in HF risk prediction, models need to be trained and evaluated on diverse populations with extensive deep phenotypes. On the other hand, there are some problems related to data sharing and the aggregation of sensitive healthcare information, as the protection of personal information is of the utmost importance. This problematic is materialized in General Data Protection Regulation (GDPR), which through various means, including data minimization, strives to limit sharing and storage of sensitive information. However, without employing the full potential of high-quality data collected globally, we will end up with numerous ML models struggling to generalize and therefore deliver clinical value. To overcome this issue, the use of federated learning (FL) could be an alternative (18).

### Federated learning

The objective of FL is to train ML models without the requirement to collect data in a central place. FL algorithms extract only the generally applicable clinical, behavioral, and physiological characteristics, without the requirement for sensitive personal information. However, there is a need for user-friendly FL tools to perform such analyses in a practical way. Such tools could allow not only federated learning (privacy-preserving collaborative model training) but also federated analysis which could supercharge the scientific progress in the CV field (and others). Similarly, the integration of deep phenotypes and the temporal domain will require specific tools for clinical practice.

### AI-based HF risk stratification tool in clinical practice

To effectively deploy a validated and robust predictive model in clinical practice, integration into the clinical workflow must be as seamless as possible. This could require integrating information from the electronic health records (EHR). However, it might be notoriously difficult, e.g., in the European settings, as there is currently a limited interoperable infrastructure. The use of EHR should be human-centric with individuals having full control over the use of their data. FL is compatible with this approach by protecting personal data. One approach to overcoming the heterogeneity of the healthcare systems is to connect systems through a system-specific bridge (adapter). Another approach is to use deep learning, which learns feature representations directly from the EHR data. This system can then transform the unstructured data from different systems into a structure that can be pipelined into another ML algorithm. These systems should be however open to ensure trust and fairness and to facilitate integration into other systems. Additionally, these systems might raise questions regarding reliability and fairness. With many current scores, there are online calculators allowing easy-to-use estimation of individual risk. However, use in the clinical setting should be cautious, as data privacy policy is not always clear and could be a possible target of 3rd-party malware attacks (the browser is more vulnerable). Therefore, an AI-based app would serve as a more secure way to handle personal data and would be more robust (e.g., working under internet connectivity disruptions). Independently on the data input method, the user experience (UX) needs to be user-friendly and informative to aid in doctor-patient communication and to provide the benefit of improved accuracy of ML-based predictive models.

## Conclusion

With a greater number and variety of training cohorts, the model learns a wider range of specific individual health characteristics. Flexible ML algorithms as well as the stacking methods can be used to capture these diverse distributions and produce more precise, personalized models for the prediction of adverse events.

## Data availability statement

The data analyzed in this study is subject to the following licenses/restrictions: The use of database should be approved by the PI of the HOMAGE study. Requests to access these datasets should be directed to Prof. Faiez Zannad, f.zannad@chru-nancy.fr.

## Ethics statement

The studies involving human participants were reviewed and approved by Ethics Committee Research UZ/KU Leuven. The patients/participants provided their written informed consent to participate in this study.

## Author contributions

FS and TK: design the sub-analysis and drafted the manuscript. TK: HOMAGE database was maintained at the Research Unit Hypertension and Cardiovascular Epidemiology in Leuven, Belgium. FS: wrote the code and performed the statistical analysis. EN and NC: provided critical revisions, read, and approved the final version of the manuscript.

## Funding

The Research Unit Hypertension and Cardiovascular Epidemiology currently received grants from Internal Funds KU Leuven (C24M/21/025) and the Research Foundation Flanders (FWO Grants 1225021N, 1S07421N, and G0C5319N).

## Conflict of interest

The authors declare that the research was conducted in the absence of any commercial or financial relationships that could be construed as a potential conflict of interest.

## Publisher's note

All claims expressed in this article are solely those of the authors and do not necessarily represent those of their affiliated organizations, or those of the publisher, the editors and the reviewers. Any product that may be evaluated in this article, or claim that may be made by its manufacturer, is not guaranteed or endorsed by the publisher.

## Footnotes

^1^ https://github.com/hcve/incidence-hf
