# Supplementary material for: Improving predictive performance in incident heart failure using machine learning and multi-center data
Source: Front Cardiovasc Med. 2022 Oct 18;9:1011071. doi: 10.3389/fcvm.2022.1011071 (PMC9623026; doi:10.3389/fcvm.2022.1011071)

Improving predictive performance in incident heart failure prediction using machine learning and multi-center data

Supplementary Material

**Supplemental Table 1.** Cohort characteristics.

|  | **ASCOT (n=19253)** | **FLEMENGHO (n=1101)** | **HEALTHABC (n=2935)** | **HVC (n=436)** | **PREDICTOR (n=1463)** | **PROSPER (n=5166)** |
| --- | --- | --- | --- | --- | --- | --- |
| **Administrative** |  |  |  |  |  |  |
| Female, n (%) | 4,514 (23) | 579 (53) | 1,528 (52) | 194 (44) | 718 (49) | 2,615 (51) |
| Age, y | 63.0 ± 8.48 | 55.2 ± 12 | 73.6 ± 2.87 | 61.5 ± 10.9 | 72 ± 3.91 | 74.6 ± 2.73 |
| Body weight, kg | 84.6 ± 15.5 | 76.7 ± 15.2 | 75.6 ± 15 | 81.8 ± 18 | 72.8 ± 13.3 | 73.8 ± 13.4 |
| Body height, cm | 171 ± 8.97 | 168 ± 9.19 | 166 ± 9.4 | 170 ± 9.8 | 166 ± 8.57 | 166 ± 9.38 |
| BMI, kg/m² | 28.7 ± 4.57 | 27.0 ± 4.48 | 27.3 ± 4.8 | 28.1 ± 5.52 | 26.4 ± 4.12 | 26.9 ± 4.19 |
| Current/Ex-smoker, n (%) | 13,044 (68) | 662 (60) | 1,627 (56) | 234 (61) | 742 (50) | 1,456 (28) |
| Alcohol intake, n (%) | 14,292 (74) | 421 (38) | 1,460 (50) | — | 859 (59) | 2,897 (56) |
| **Blood pressure** |  |  |  |  |  |  |
| SBP, mmHg | 164 ± 18 | 132 ± 17.4 | 136 ± 20.9 | 149 ± 23 | 138 ± 16.9 | 154 ± 21.8 |
| DBP, mmHg | 94.7 ± 10.4 | 81.6 ± 9.32 | 71.4 ± 11.7 | 84.5 ± 12 | 80.8 ± 9.4 | 83.8 ± 11.5 |
| Heart rate, BPM | 71.9 ± 12.6 | 63.6 ± 9.22 | 65.3 ± 11.1 | 73.9 ± 15.1 | 70.3 ± 11.2 | 66.3 ± 11.7 |
| Pulse pressure, mmHg | 69.4 ± 16.6 | 50.2 ± 14.9 | 64.5 ± 18 | 64.5 ± 18.6 | 57.4 ± 13 | 70.4 ± 18.1 |
| **Medical history** |  |  |  |  |  |  |
| Diabetes, n (%) | 5,145 (27) | 57 (5) | 431 (15) | 70 (16) | 241 (17) | 563 (11) |
| CVD, n (%) | 8,276 (43) | 102 (9) | 740 (26) | 305 (70) | 392 (27) | 2,212 (43) |
| CAD, n (%) | 5,284 (27) | 54 (5) | 637 (22) | 70 (16) | 110 (8) | 1,634 (32) |
| PAD, n (%) | 1,199 (6) | 27 (2) | 136 (5) | 45 (10) | 0 (0) | 112 (2) |
| A-fib, n (%) | 230 (1) | 21 (2) | — | 37 (8) | 0 (0) | — |
| CVA, n (%) | 2,113 (11) | 23 (2) | 221 (8) | 41 (9) | 0 (0) | 552 (11) |
| **Medication** |  |  |  |  |  |  |
| Treated for HT, n (%) | 15,588 (81) | 322 (29) | 1,545 (53) | 342 (78) | 742 (51) | 3,781 (73) |
| ACE blockers, n (%) | 4,972 (32) | 64 (6) | 413 (14) | 116 (27) | 299 (20) | 838 (16) |
| CC blockers, n (%) | 5,513 (35) | 67 (6) | 660 (23) | 116 (27) | 235 (16) | 1,301 (25) |
| Intake of diuretics, n (%) | 5,488 (35) | 119 (11) | 714 (24) | 119 (27) | 222 (15) | 2,057 (40) |
| Beta-blokcers, n (%) | 6,156 (39) | 185 (17) | 364 (12) | 235 (54) | 214 (15) | 1,330 (26) |
| AR blockers, n (%) | 1,218 (8) | 47 (4) | 61 (2) | 117 (27) | 298 (20) | 103 (2) |
| Lipid-lowering, n (%) | 2,050 (11) | 194 (18) | 403 (14) | 240 (55) | — | — |
| **ECG** |  |  |  |  |  |  |
| Sokolow-Lyon index, mm | 24.2 ± 8.22 | 21.1 ± 7.08 | — | — | — | — |
| QRS duration, ms | 94.3 ± 15.1 | 92.9 ± 14.1 | 93 ± 17.9 | 92.5 ± 16.9 | — | — |
| Cornell index, mm x msec | 1710 ± 808 | 1110 ± 669 | — | — | — | — |
| **Biochemical** |  |  |  |  |  |  |
| Blood glucose, mmol/L | 6.24 ± 2.12 | 4.89 ± 0.764 | 5.77 ± 1.9 | 6.15 ± 2.21 | 5.79 ± 1.53 | 5.48 ± 1.47 |
| Total cholesterol, mmol/L | 5.91 ± 1.08 | 5.21 ± 0.953 | 5.26 ± 0.992 | 5.32 ± 1.31 | 5.3 ± 1.02 | 5.67 ± 0.903 |
| LDL-cholesterol, mmol/L | 3.78 ± 0.966 | 3.16 ± 0.843 | 3.16 ± 0.896 | 3.31 ± 1.12 | 3.31 ± 0.894 | 3.79 ± 0.797 |
| HDL-cholesterol, mmol/L | 1.3 ± 0.365 | 1.45 ± 0.38 | 1.41 ± 0.441 | 1.25 ± 0.412 | 1.36 ± 0.342 | 1.28 ± 0.349 |
| Triglycerides, mmol/L | 1.84 ± 1 | 1.29 ± 0.723 | 1.51 ± 0.849 | 1.81 ± 1.21 | 1.32 ± 0.631 | 1.54 ± 0.697 |
| Serum creatinine, mmol/L | 98.7 ± 16.8 | 80.2 ± 16.8 | 92.9 ± 36.1 | 95 ± 64.6 | 83.1 ± 21.5 | 101 ± 22 |
|  |  |  |  |  |  |  |

SBP-Systolic blood pressure, PAD-peripheral artery disease, CVD-Cardiovascular disease, CAD-coronary artery disease, A-fib-atrial fibrillation, ACE-angiotensin converting enzyme, HT-hypertension, CC-calcium channel, AR-angiotensin II receptor, SBP-Systolic blood pressure, DBP-Diastolic blood pressure, BPM-beats per minute**Supplemental Table 2**. Missing values in cohorts. SBP-Systolic blood pressure, PAD-peripheral artery disease, CVD-Cardiovascular disease, CAD-coronary artery disease, A-fib-atrial fibrillation, ACE-angiotensin converting enzyme, HT-hypertension, CC-calcium channel, AR-angiotensin II receptor, SBP-Systolic blood pressure, DBP-Diastolic blood pressure, BPM-beats per minute

|  | **ASCOT (n=19253)** | **FLEMENGHO (n=1101)** | **HEALTHABC (n=2935)** | **HVC (n=436)** | **PREDICTOR (n=1463)** | **PROSPER (n=5166)** | |
| --- | --- | --- | --- | --- | --- | --- | --- |
| **Administrative** |  |  |  |  |  |  | |
| Female, n (%) |  |  |  |  |  |  | |
| Age, y |  |  |  |  |  |  | |
| Body weight, kg |  |  |  | 15.4 | 0.3 |  | |
| Body height, cm |  |  |  | 11.2 | 0.5 | 0.0 | |
| BMI, kg/m² |  |  |  | 16.1 | 0.8 | 0.0 | |
| Current/Ex-smoker, n (%) |  |  | 0.1 | 11.7 | 0.1 |  | |
| Alcohol intake, n (%) |  |  | 0.5 | 100.0 | 0.1 |  | |
| **Blood pressure** |  |  |  |  |  |  | |
| SBP, mmHg |  |  |  |  |  |  | |
| DBP, mmHg |  |  |  |  | 0.1 |  | |
| Heart rate, BPM |  |  | 0.1 | 1.1 | 0.1 | 2.1 | |
| Pulse pressure, mmHg |  |  |  |  | 0.1 |  | |
| **Medical history** |  |  |  |  |  |  | |
| Diabetes, n (%) |  |  | 0.1 |  | 0.8 |  | |
| CVD, n (%) |  |  | 1.9 |  | 0.2 |  | |
| CAD, n (%) |  |  | 1.5 |  |  |  | |
| PAD, n (%) |  |  | 2.3 |  |  |  | |
| A-fib, n (%) | 0.7 |  | 100.0 |  |  | 100.0 | |
| CVA, n (%) |  |  | 1.0 |  |  |  | |
| **Medication** |  |  |  |  |  |  | |
| Treated for HT, n (%) |  |  | 0.3 |  |  |  | |
| ACE blockers, n (%) | 19.0 |  | 0.3 |  |  |  | |
| CC blockers, n (%) | 19.0 |  | 0.3 |  |  |  | |
| Intake of diuretics, n (%) | 19.0 |  |  |  |  |  | |
| Beta-blokcers, n (%) | 19.0 |  | 0.3 |  |  |  | |
| AR blockers, n (%) | 19.0 |  | 0.3 |  |  |  | |
| Lipid-lowering, n (%) |  |  | 0.3 |  | 100.0 | 100.0 | |
| **ECG** |  |  |  |  |  |  | |
| Sokolow-Lyon index, mm | 2.1 | 1.2 | 100.0 | 100.0 | 100.0 | 100.0 | |
| QRS duration, ms | 1.2 | 1.2 | 0.3 | 1.1 | 100.0 | 100.0 | |
| Cornell index, mm x msec | 2.9 | 1.2 | 100.0 | 100.0 | 100.0 | 100.0 | |
| **Biochemical** |  |  |  |  |  |  | |
| Blood glucose, mmol/L | 9.5 | 0.1 | 1.0 | 8.5 | 0.5 | 0.4 | |
| Total cholesterol, mmol/L |  | 0.1 | 1.1 | 1.8 | 0.4 |  | |
| LDL-cholesterol, mmol/L | 11.2 | 0.3 | 2.3 | 2.5 | 1.1 |  | |
| HDL-cholesterol, mmol/L |  | 0.1 | 1.2 | 2.1 | 0.8 |  | |
| Triglycerides, mmol/L | 9.3 | 0.1 | 1.1 | 1.8 | 0.5 |  | |
| Serum creatinine, mmol/L | 31.9 | 0.1 | 0.9 | 1.4 | 0.5 | 0.1 |  |

SBP-Systolic blood pressure, PAD-peripheral artery disease, CVD-Cardiovascular disease, CAD-coronary artery disease, A-fib-atrial fibrillation, ACE-angiotensin converting enzyme, HT-hypertension, CC-calcium channel, AR-angiotensin II receptor, SBP-Systolic blood pressure, DBP-Diastolic blood pressure, BPM-beats per minute**Supplemental Table 3**. Characteristics of CV events in the studied cohorts.

|  | **ASCOT (n=19253)** | **FLEMENGHO (n=1101)** | **HEALTHABC (n=2935)** | **HVC (n=436)** | **PREDICTOR (n=1463)** | **PROSPER (n=5166)** |
| --- | --- | --- | --- | --- | --- | --- |
| **Cardiovascular death** |  |  |  |  |  |  |
| Events per 1000 py | 5.70 | 1.92 | 5.53 | 13.90 | 3.52 | 14.56 |
| Incidence | 3.1% | 1.8% | 5.9% | 2.3% | 1.4% | 4.7% |
| Events | 605 | 20 | 173 | 10 | 20 | 243 |
| Median follow-up (years) | 5.6 | 10.3 | 12.9 | 1.1 | 4.0 | 3.3 |
| **Non-fatal heart failure** |  |  |  |  |  |  |
| Events per 1000 py | 2.30 | 2.40 | 19.51 | 15.75 | 5.50 | 11.59 |
| Incidence | 1.3% | 1.6% | 19.6% | 2.5% | 2.1% | 3.7% |
| Events | 243 | 18 | 574 | 11 | 31 | 191 |
| Median follow-up (years) | 5.6 | 6.9 | 12.0 | 1.0 | 3.9 | 3.3 |

**Supplemental Figure 1.** Calibration plots of trained models (SGB and CoxNet). Both models showed good calibration, but SGB overestimates risk for some low-risk individuals more.


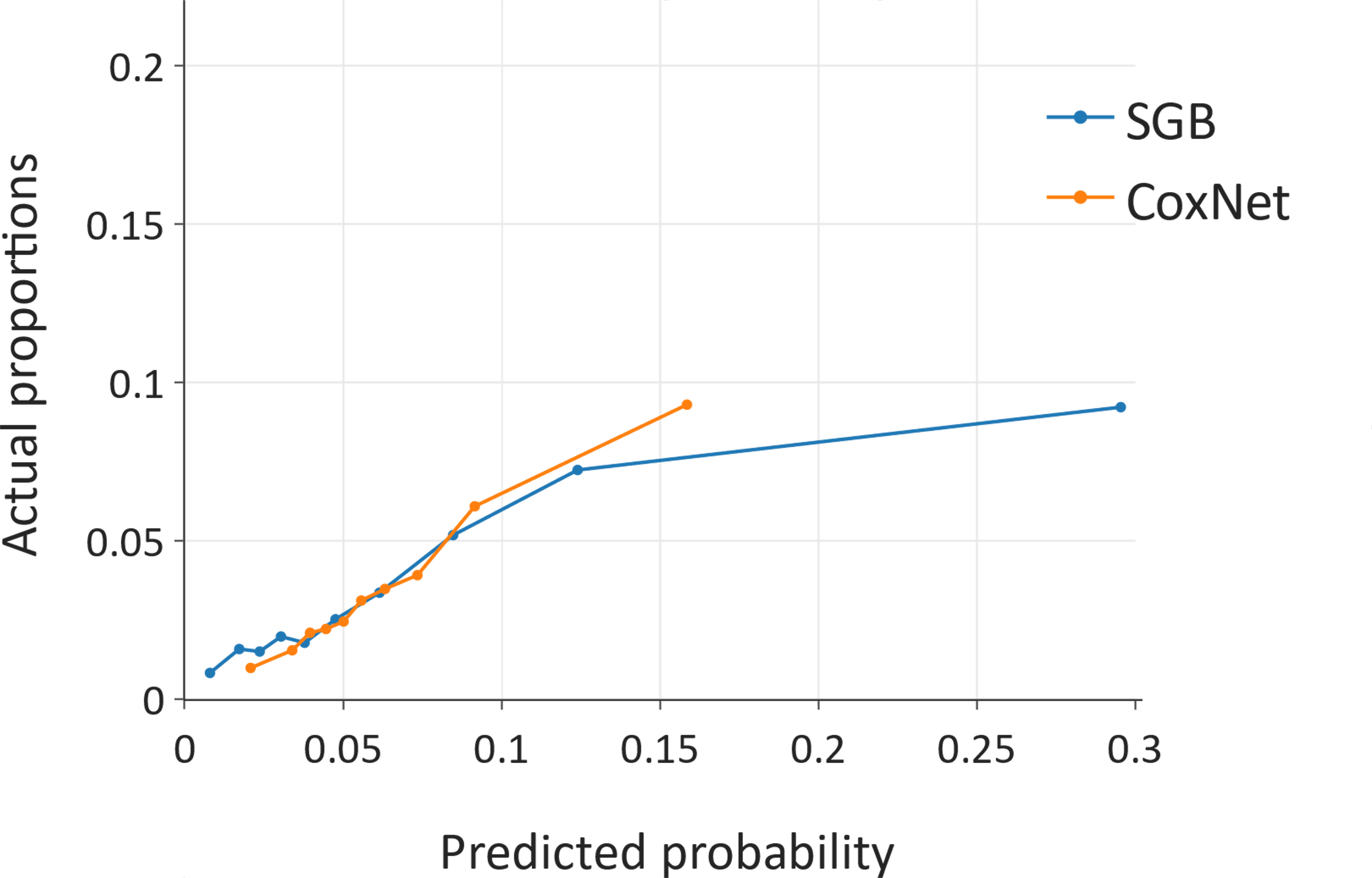

Supplement: Supplementary file 1 [file Data_Sheet_1.docx]
